# Drought stress in maize causes differential acclimation responses of glutathione and sulfur metabolism in leaves and roots

**DOI:** 10.1186/s12870-016-0940-z

**Published:** 2016-11-09

**Authors:** Nisar Ahmad, Mario Malagoli, Markus Wirtz, Ruediger Hell

**Affiliations:** 1 Centre for Organismal Studies Heidelberg, Heidelberg University, Im Neuenheimer Feld 360, 69120 Heidelberg, Germany; 2 University of Science & Technology Bannu, Bannu, Pakistan; 3 Department of Agronomy, Food, Natural Resources, Animals and Environment, University of Padova, Padova, Italy

**Keywords:** *Zea mays*, Cysteine, Sulfate assimilation, Flux analysis, Glutathione synthesis, Reactive oxygen species

## Abstract

**Background:**

Drought is the most important environmental stress that limits crop yield in a global warming world. Despite the compelling evidence of an important role of oxidized and reduced sulfur-containing compounds during the response of plants to drought stress (e.g. sulfate for stomata closure or glutathione for scavenging of reactive oxygen species), the assimilatory sulfate reduction pathway is almost not investigated at the molecular or at the whole plant level during drought.

**Results:**

In the present study, we elucidated the role of assimilatory sulfate reduction in roots and leaves of the staple crop maize after application of drought stress. The time-resolved dynamics of the adaption processes to the stress was analyzed in a physiological relevant situation –when prolonged drought caused significant oxidation stress but root growth should be maintained. The allocation of sulfate was significantly shifted to the roots upon drought and allowed for significant increase of thiols derived from sulfate assimilation in roots. This enabled roots to produce biomass, while leaf growth was stopped. Accumulation of harmful reactive oxygen species caused oxidation of the glutathione pool and decreased glutathione levels in leaves. Surprisingly, flux analysis using [^35^S]-sulfate demonstrated a significant down-regulation of sulfate assimilation and cysteine synthesis in leaves due to the substantial decrease of serine acetyltransferase activity. The insufficient cysteine supply caused depletion of glutathione pool in spite of significant transcriptional induction of glutathione synthesis limiting *GSH1*. Furthermore, drought impinges on transcription of membrane-localized sulfate transport systems in leaves and roots, which provides a potential molecular mechanism for the reallocation of sulfur upon prolonged water withdrawal.

**Conclusions:**

The study demonstrated a significant and organ-specific impact of drought upon sulfate assimilation. The sulfur metabolism related alterations at the transcriptional, metabolic and enzyme activity level are consistent with a promotion of root growth to search for water at the expense of leaf growth. The results provide evidence for the importance of antagonistic regulation of sulfur metabolism in leaves and roots to enable successful drought stress response at the whole plant level.

**Electronic supplementary material:**

The online version of this article (doi:10.1186/s12870-016-0940-z) contains supplementary material, which is available to authorized users.

## Background

Plants encounter during their life cycle various environmental stresses that adversely affect growth and development. Drought, salinity and extreme temperature are the abiotic stresses that are responsible for up to 50–70 % decline in major crop production [1]. Water shortage is the single one factor for plant growth that ultimately causes reduction in crop yield more than any other stress condition [2]. Maize is cultivated in over 170 million hectares in the world and is considered the second most important staple crop (FAO statistical database, http://faostat3.fao.org/home/E). Thus, understanding the drought adaptation of maize is crucial and a prerequisite to sustain plant productivity.

The root is the primary organ that responds at early stages to decreases in soil water status. Abscisic acid (ABA) plays a key role in root-to-shoot signaling and in the partial or complete stomatal closure to reduce transpiration [3]. Recently, sulfate has been shown to promote ABA synthesis [4] and was found to be transported earlier than ABA from the root to the shoot upon drought stress [5]. In addition to stomata closure, drought-induced ABA triggers many physiological responses like glycinebetaine production and root growth of maize plants [6]. During drought the root system is usually elongated to improve uptake of water from the soil, whereas the shoot growth is inhibited [7]. In maize, drought stress-induced promotion of root growth is supposed to be affected by ABA-responsive *miR169* family members that control general transcription factors of the NF-YA type [8]. In addition to the general promotion of root growth also root architecture is affected upon drought [9]. Drought and ABA inhibit lateral root formation [10]. In combination with the general increase of root growth, this facilitates growth of the primary root into deeper soil areas. Field studies clearly demonstrate that deep-rooted plants perform better than shallow-rooted genotypes under drought stress due to better acquisition of water in deeper areas of the soil profile [9]. Recently, ABA-induced down-regulation of the NatA complex has been evidenced to mediate stomata closure and decreased lateral root formation in Arabidopsis. Consequently, genetically engineered plants with decreased NatA activity are highly drought tolerant [11]. Taken together, these evidences demonstrate the importance of developmental plasticity for an adequate whole plant response to restricted water access.

At the cellular level, limited water supply enhances the production of reactive oxygen species (ROS), particularly in chloroplasts, mitochondria and peroxisomes. While low steady-state levels of ROS can be used by cells to monitor stress, concentrations that exceed the cellular antioxidant defense systems can become deleterious and ultimately lead to cell death [12, 13]. These defense systems include enzymes such as superoxide dismutase, catalase, and peroxidases and the ascorbate-glutathione cycle. In this cycle H_2_O_2_ is reduced to H_2_O via ascorbate and reduced glutathione (GSH) and as a result oxidized glutathione (GSSG) is formed which is recycled back to GSH by the action of glutathione reductase (GR) using NADPH as reductant (reviewed in [14]). Enhanced GR activities in response to drought stress serve to maintain the ratio of reduced to oxidized glutathione and thus the redox potential of glutathione, and have been reported from numerous plant species including maize ([12], reviewed in [15]). GR is so essential for the survival of cells that it is present in plastids, mitochondria, peroxisomes and the cytosol and NADPH-dependent thioredoxin reductases have evolved as back-up systems [16]. Accumulation of antioxidants and ROS scavengers are believed to be part of evolutionary traits towards tolerance to severe drought [17]. In fact, engineered over-expression of the antioxidant enzymes resulted in enhanced tolerance to drought, salt or osmotic stress in several plant species [13].

In addition to GR activity the *de novo* synthesis of glutathione can support maintenance of the GSH/GSSG ratio as has been observed for several environmental factors leading to oxidative stress [18–21]. Increases in the pool of total glutathione might be partially masked by the degradation of GSSG in the vacuole to recycle cysteine [22]. Glutathione biosynthesis is a two-step process. First, the synthesis of γ-glutamylcysteine (γ-EC) takes place from cysteine and glutamate catalyzed by GSH1. In the second step, GSH is formed by the addition of glycine to γ-EC catalyzed by GSH2. GSH1 activity is rate limiting in GSH biosynthesis and is feedback inhibited by GSH [23]. Cysteine with its sulfhydryl moiety is the major functional component in glutathione. It is the endproduct of the assimilatory sulfate reduction pathway and is synthesized by the enzymes serine acetyltransferase (SERAT) and *O*-acetylserine (thiol) lyase (OAS-TL) via the intermediate *O*-acetylserine (OAS). Sulfide is generated in plastids from sulfate in three subsequent reactions that are catalyzed by ATP sulfurylase (ATPS), adenosine-phosphosulfate reductase (APR) and sulfite reductase (SiR). Sulfate is taken up from the soil and distributed within the plant by sulfate transporters (SULTR) in the plasmalemma. The sulfur assimilation pathway and its regulation has been well investigated in *Arabidopsis thaliana* [24], mostly under environmental sulfate deficiency. Maize has been much less analyzed with respect to sulfur uptake and metabolism although the biochemical steps are highly conserved [25, 26]. Major differences to the C3 plant Arabidopsis were associated with the compartmentation of C4 metabolism in maize leaves. The sulfate reduction pathway is almost exclusively localized in the chloroplasts of bundle sheath cells but not of mesophyll cells, whereas glutathione can be synthesized in both cell types [27]. Consequently, cysteine but not glutathione like in C3 plants is a major intercellular transport form of reduced sulfur [28]. These differences between C4 and C3 plants seem to extend to regulatory mechanisms since cysteine but not glutathione has been found to control of the nutritional status of maize roots [29].

The role of the sulfate assimilation pathway towards glutathione synthesis in response to drought-induced oxidative stress has hardly been investigated. The response of primary sulfur metabolism to prolonged drought stress was therefore investigated in an integrative study of leaf and root processes at the levels of physiology, metabolites and gene expression. The results reveal that the increasing limitation of sulfate in leaves during drought is insufficiently counteracted by differential expression of key genes of sulfate transport and glutathione metabolism, leading to lowered flux in the pathway, enhanced oxidative stress and growth arrest. In contrast, the roots have sufficient sulfate available to cope with the oxidative stress due to effective maintenance of the glutathione redox system, thereby contributing to enhanced root growth and resistance to water limited conditions.

## Results

### Impact of drought on maize

**Fig. 1:**
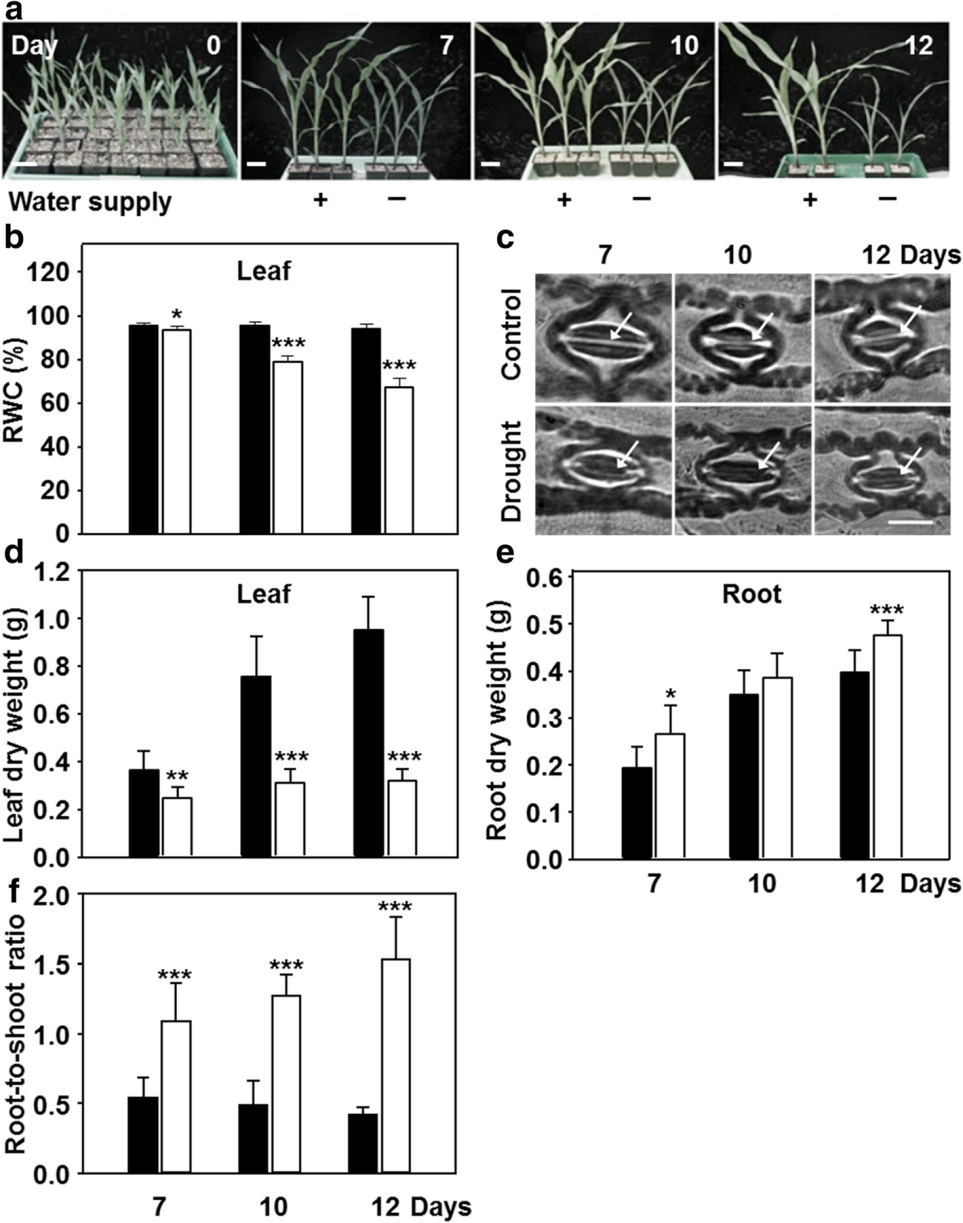
Developmental response of maize to restricted water supply. **a** Growth phenotypes of maize hybrid Severo grown on vermiculite as described in materials in presence (*black*) or absence (*white*) of continuous water supply for up to 12 days (Scale bar = 8 cm). **b**, **d**, **e** Relative water content (**b**), dry weight (**d**, **e**) of leaves (**a**) and roots (**b**, **e**) from plants shown in **a**. **c** Stomata of control and drought-stressed maize leaves at indicated time points. Arrows indicate the pore. Scale bar = 20 μm. **f** Root-to-shoot ratio determined from data shown in **d** and **e**. Data are means ± SD of eight individual replicates. Asterisks indicates statistical differences as determined by the unpaired *t*-test (*, 0.05 ≥ *p* > 0.01; **, 0.01 ≥ *p* > 0.001; ***, *p* ≤ 0.001)

Maize plants were grown for 2 weeks on vermiculite medium as it facilitates the harvest of roots as compared to soil-grown plants and then subjected to a time course of drought stress for 7, 10 and 12 days (Fig. 1a). The imposition of drought to maize plants severely decreased relative water content (RWC) of leaf from day 10 on, while the control plants remained at 96 % RWC. Water withdrawal for 7 d had a significant but small effect on the RWC (Fig. 1b). Stomata closure is one of the first responses of plants to water shortage to minimize water loss due to transpiration. In comparison to control, drought-treated plants exhibited decreases in stomatal aperture at each time point, implicating lowered vascular water transport (Fig. 1c, Additional file 1: Figure S1). The growth response to drought was characterized by determination of dry weight. Dry weight accumulation of control leaves increased linearly but stopped almost completely from day 7 of drought onwards. Roots in contrast continued dry weight accumulation under water-stressed conditions (Fig. 1d, e), establishing the characteristic drought response of increased root-to-shoot ratio. From day 7 to 12 of drought the root-to-shoot ratio increased linearly in maize indicating significant reallocation of resources from the shoot to the root and an active root metabolism (Fig. 1f). Reapplication of water at day 12 was able to rescue drought stressed maize plants, defining these experimental conditions as physiologically realistic for environmental drought stress.

### Drought stress and oxidative stress markers

**Fig. 2:**
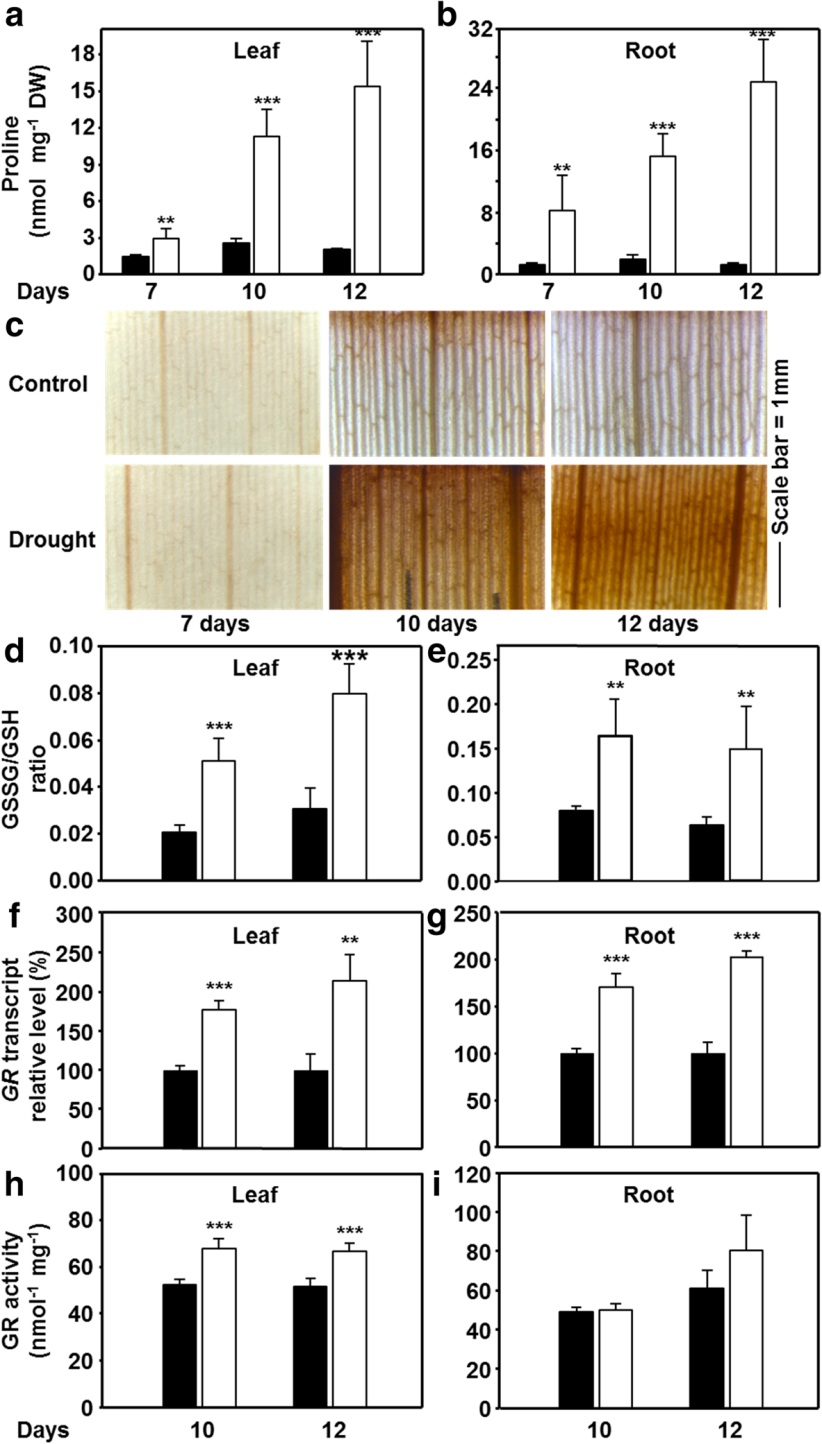
Impact of drought on stress markers and reactive oxygen species formation in leaves and roots of maize. **a**-**b** Proline steady state levels in leaves (**a**) and roots (**b**) in control conditions (*black*) and after restriction of water supply (*white*) for indicated time points (*n* = 5). **c** In situ staining of hydrogen peroxide formation in leaves of drought-stressed maize (*n* = 3). **d**-**e** Oxidation of the glutathione pool (GSSG/GSH ratio) in leaves and roots of drought-stressed maize (*n* = 5). **f**-**i** Impact of drought stress on transcription (**f**, **g**, *n* = 3) and enzymatic activity (**h**, **i**, *n* = 4) of glutathione reductase (GR) in leaves (**f**, **h**) and roots (**g**, **i**) of maize. Data are means ± SD of three to five individual replicates. Asterisks indicates statistical differences as determined by the unpaired *t*-test (*, 0.05 ≥ *p* > 0.01; **, 0.01 ≥ *p* > 0.001; ***, *p* ≤ 0.001)

The drought stress response of maize plants was further characterized with respect to metabolic changes with the aim to identify an early stage of comprehensive acclimation responses upon appearance of ROS. Proline accumulation is reported in maize leaves and roots upon water scarcity, and can be used as marker for drought stress [30]. The proline level was about doubled in leaves after 7 d of drought and increased 4- to 7-fold in the following 5 days compared to well-watered control plants. Roots as primary site of drought reception responded much stronger with 8-fold increase at day 7 and to up 25-fold increase of proline level after 12 days of drought (Fig. 2a, b). This indicates the proper onset of drought-induced stress both in leaves and roots in maize.

The production of ROS started later according to visualization of H_2_O_2_ levels as marker for oxidative stress. 7 d of drought did not affect H_2_O_2_ level compared to control. The intensity of H_2_O_2_ staining was much more pronounced in all analyzed leaf areas after 10 and 12 d of stress showing high H_2_O_2_ amounts were produced in response to drought (Fig. 2c). Since 7 d of water withdrawal did not increase H_2_O_2_ production and only slightly affected the leaf RWC, the 10 and 12 d time points were selected for all further analyses.

Consistent with the observed H_2_O_2_ accumulation, the oxidized (GSSG) to reduced (GSH) glutathione ratio in leaves was significantly increased by 2.5 and 2.6-fold after 10 and 12 days of drought, respectively (Fig. 2d). In well-watered plants the roots showed already a more oxidized condition with higher GSSG/GSH ratio compared to leaves. However, this ratio additionally shifted 2-to 2.3-fold towards the oxidized state upon drought, indicating that roots also underwent severe oxidative stress (Fig. 2e).

Glutathione reductase (GR) regenerates GSH at the expense of NADPH during ROS detoxification via the ascorbate-glutathione cycle. A blast search using the maize database (maizegenome.org) revealed only one GR (GenBank accession no. AJ006055) based on protein sequence similarity shared with Arabidopsis GRs (Additional file 2: Figure S2). The *GR* transcript was up-regulated both in leaves (1.7 and 2.2-fold) and roots (1.7- and 2-fold) after 10 and 12 days of drought, respectively (Fig. 2f, g). A significant increase in total GR enzyme activity (25–30 %) was observed in leaf relative to control (Fig. 2h), while small increases of root GR activity were not statistically significant (Fig. 2i). Together these results demonstrate that when water was withheld for 10 and 12 days the leaves as well as the roots suffered from oxidative stress that challenged glutathione metabolism.

### Effects of drought on glutathione biosynthesis

**Fig. 3:**
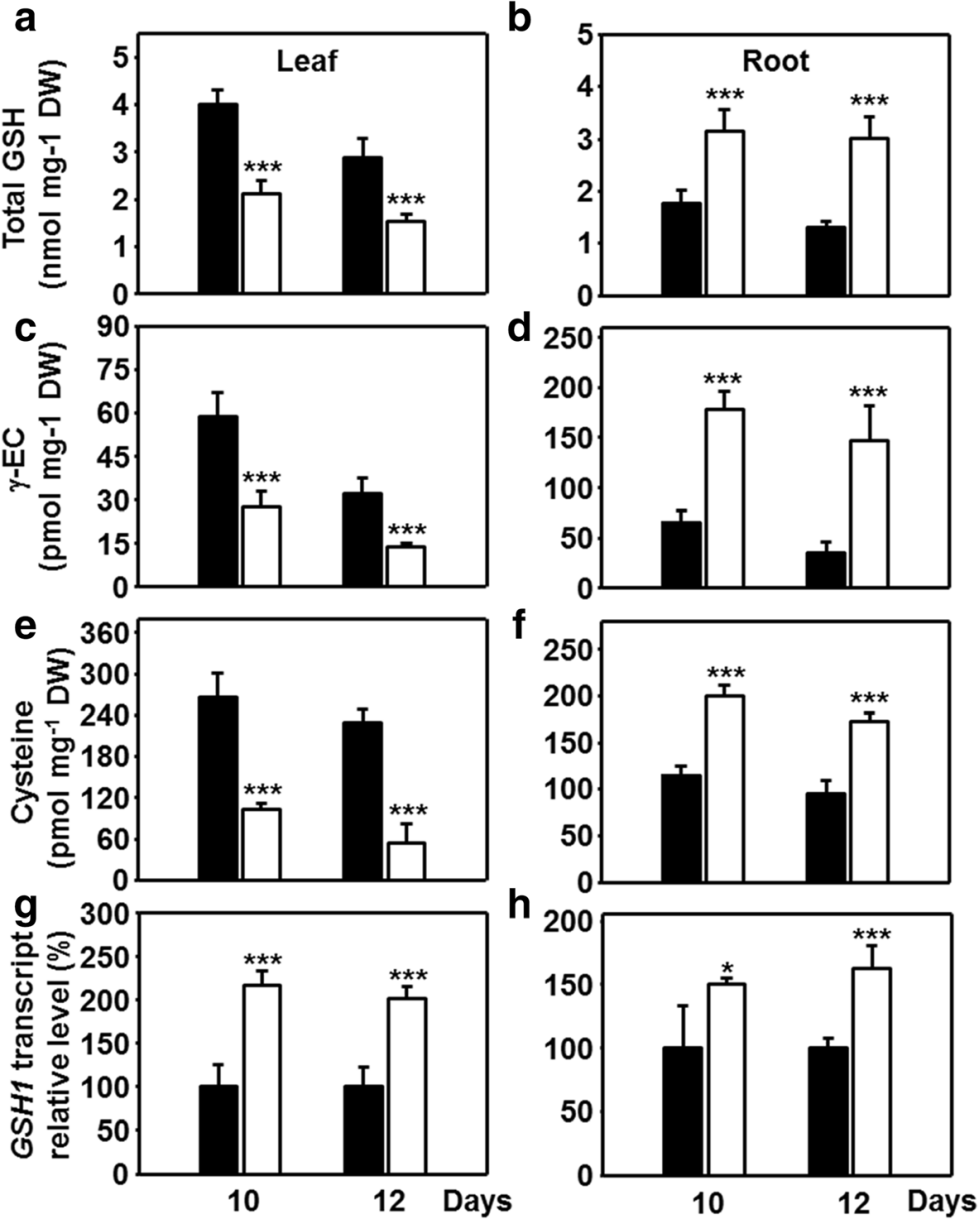
Glutathione production in leaves and roots of drought-stressed maize. A-F) Steady state levels of glutathione (**a**, **b**), the glutathione precursor γ-EC (**c**, **d**) and cysteine (**e**, **f**) in leaves (**a**, **c**, **e**) and roots (**b**, **d**, **f**) of maize plants with sufficient (*black*) and restricted (*white*) water supply. **g**, **h** Relative transcript levels of the γ-EC-synthase (*GSH1*) in leaves (**g**) and roots (**h**) of drought-stressed plants. Data are means ± SD of five (**a**-**f**) or three (**g**) individual replicates. Asterisks indicates statistical differences as determined by the unpaired *t*-test (*, 0.05 ≥ *p* > 0.01; **, 0.01 ≥ *p* > 0.001; ***, *p* ≤ 0.001)

**Fig. 4:**
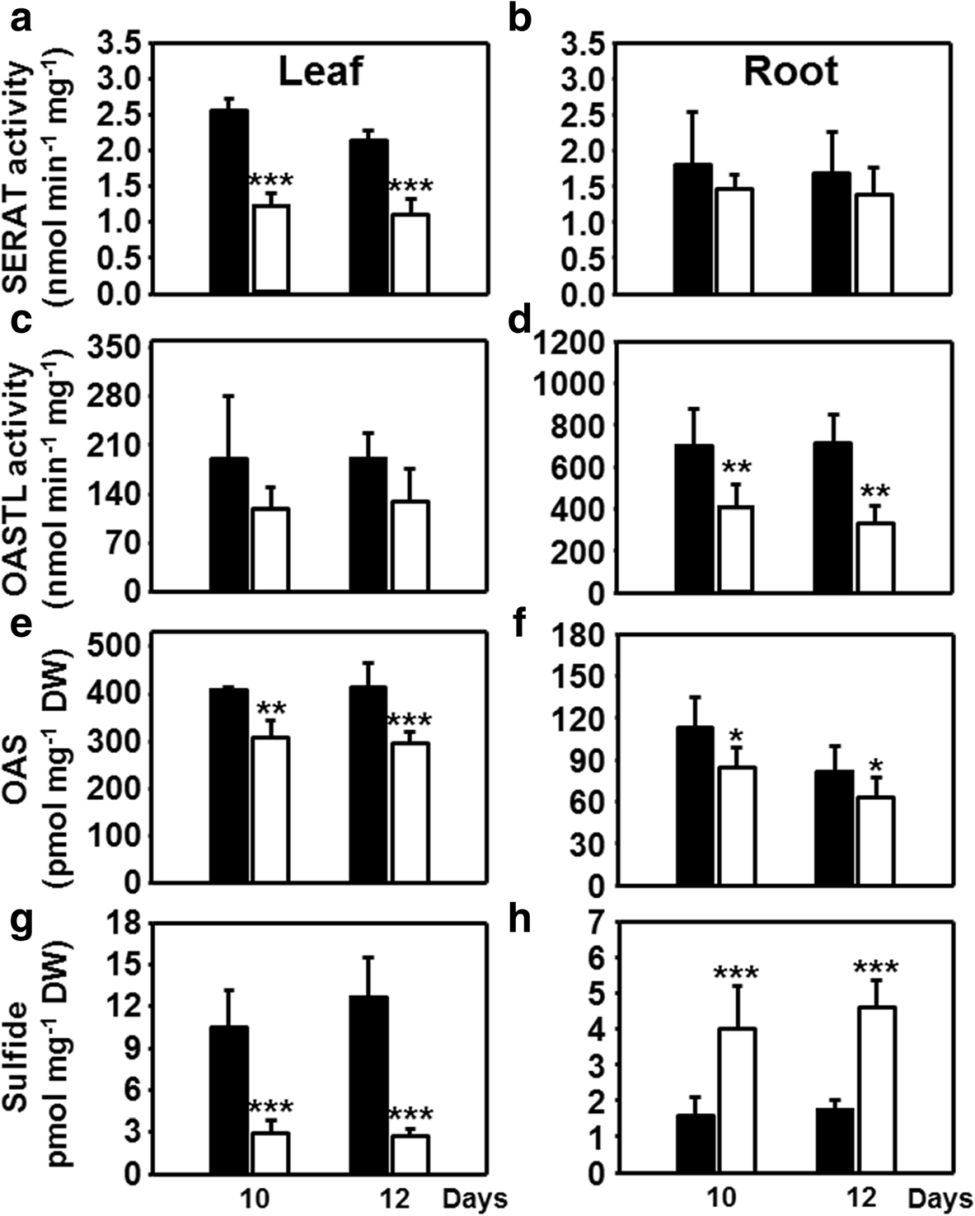
Organ-specific impact of drought stress on cysteine biosynthesis in maize. **a**-**d** Extractable enzymatic activities of serine acetyltransferase (**a**, **b**, SERAT) and *O*-acetylserine(thiol)lyase (**c**, **d**, OASTL) from leaves (**a**, **c**) and roots (**c**, **d**) of control (*black*) and drought-stressed plants (*white*). **e**-**h** Steady state levels of the cysteine precursors OAS (**e**, **f**) and sulfide (**g**, **h**) in leaves (**e**, **g**) and roots (**f**, **h**) of maize plants suffering from water restriction. Data are means ± SD of five to seven individual replicates. Asterisks indicates statistical differences as determined by the unpaired *t*-test (*, 0.05 ≥ *p* > 0.01; **, 0.01 ≥ *p* > 0.001; ***, *p* ≤ 0.001)

The alterations in the redox state of the glutathione pool were further investigated with respect to total glutathione concentrations and its biosynthetic pathway. Determination of glycine and glutamate levels in roots and leaves revealed only minor alterations upon application of drought stress (Additional file 3: Figure S3). We consequently focused on the provision of cysteine for glutathione biosynthesis, which is limiting GSH biosynthesis during the day in plants [31]. In leaves of drought-stressed maize not only the steady state level of glutathione was decreased by approximately 50 % but also those of the precursors γ- EC and cysteine to 60 and 75 %, respectively (Fig. 3a, c, e). In roots of control plants the concentration of glutathione was only about half of that in leaves. Under water scarcity, roots showed increases of total glutathione concentrations of 1.8 and 2.3-fold relative to control that even reached the levels observed in leaves of non-stressed maize plants (Fig. 3b). Correspondingly, γ-EC and cysteine contents also exhibited elevated levels of the same extent (Fig. 3d, f). The same pattern of increased levels in roots and decreased levels in leaves was also observed for sulfide (Fig. 4g, h), the primary product of sulfate reduction.

The rate limiting role of γ-glutamylcysteine ligase (*GSH1*) in GSH biosynthesis [23] prompted us to quantify mRNA abundance of *GSH1* in leaves and roots under drought. An approximately 2-fold increase in the transcript amount of *GSH1* was noted in leaves and of 1.5-fold in roots compared to controls (Fig. 3g, h). It is concluded that the drought response program operated towards enhanced glutathione biosynthesis in leaves and roots, but only in roots the availability of precursors allowed to elevate concentrations of total glutathione. The significant contribution of higher total glutathione levels to the redox potential might compensate for the modest increase of GR activity in drought-stressed roots (Fig. 2i).

In search for a mechanistic explanation of the different glutathione levels it was the availability of cysteine that distinguished the response of roots from the one in leaves. We therefore determined the activities of the enzymes of cysteine synthesis in both organs. SERAT catalyzes the rate-limiting reaction of OAS formation from serine and acetyl coenzyme A, whereas OASTL activity substitutes the acetyl group of OAS with sulfide to produce cysteine [32]. The total extractable SERAT and OASTL activities were measured in order to test if the changed cysteine contents in leaves and roots were due to drought-induced changes in enzyme activities. As observed for other plant species, total OAS-TL activity was about 50–500 times higher than SERAT activities [24, 33–36], stating that the latter catalyzes the rate-limiting reaction also in maize (Fig. 4a-d). In leaves drought treatment resulted in significantly decreased SERAT activity (Fig. 4a), lowered OAS (Fig. 4e) and sulfide concentration (Fig. 4g) compared to controls, while OASTL activity was not affected (Fig. 4c). Surprisingly drought-stressed roots did not show this overall decrease of the cysteine biosynthesis pathway: SERAT activities were maintained and sulfide levels even increased (Fig. 4b, h). Most probably the higher availability of sulfide allowed the decreased but not limiting OAS-TL activity (Fig. 4d) to convert OAS into cysteine, which is also supported by lowered OAS (Fig. 4f) and higher cysteine steady state levels (Fig. 3f). This observation is remarkable since sulfide is the endproduct of assimilatory sulfate reduction and considered to be indicative of the activity of the pathway [25, 37].

Together these results point to differential responses in roots and leaves, ultimately providing (roots), or not providing (leaves), reduced sulfur for glutathione synthesis towards detoxification of ROS and maintenance of redox potential.

### Impact of drought on sulfur accumulation and on sulfur metabolism-related gene expression

**Fig. 5:**
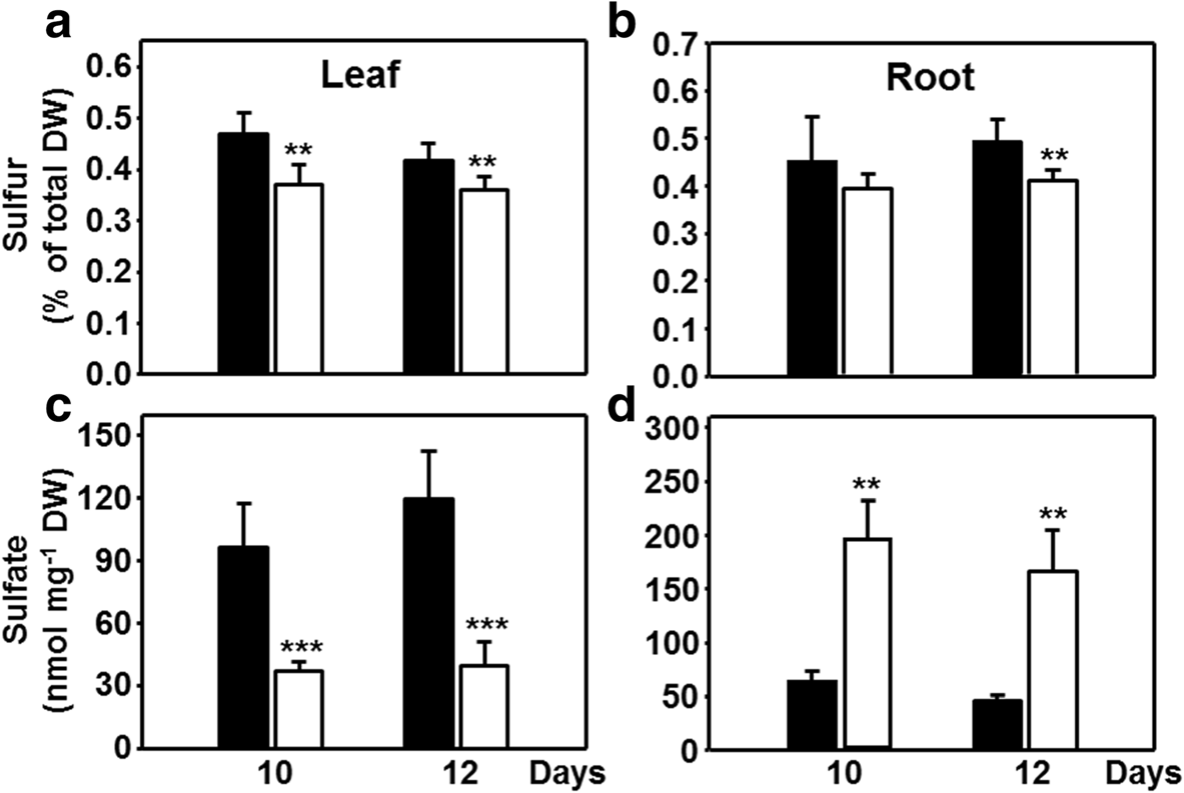
Allocation of sulfur and sulfate in drought-stressed maize. Abundance of total sulfur as percent of dry matter content (**a**, **b**) and sulfate (**c**, **d**) in leaves (**a**, **c**) and roots (**b**, **d**) of control (*black*) and drought-stressed (*white*) plants. Data are means ± SD of five to seven individual replicates. Asterisks indicates statistical differences as determined by the unpaired *t*-test (*, 0.05 ≥ *p* > 0.01; **, 0.01 ≥ *p* > 0.001; ***, *p* ≤ 0.001)

The differential response of leaves and roots to drought with respect to sulfide levels was further investigated by measuring the accumulation of total sulfur during drought stress. The total content of sulfur, expressed as % elemental S of dry weight, was significantly decreased in leaves of drought-stressed plants. It was also lowered in roots compared to well-watered controls, although significantly only after 12 d (Fig. 5a, b). However, if the amount of sulfur in roots is calculated as mg S per total root biomass (about 1.6 mg at 10 d, and 2.0 mg at 12d), the contents were unchanged between stressed and non-stressed roots. This finding, together with the enhanced growth (Fig. 1e), points to a sufficient sulfur supply of roots under drought. Interestingly, the free sulfate levels decreased 2.5-3-fold in leaves but in contrast increased about the same magnitude in roots upon drought (Fig. 5c, d). The data strongly suggest that upon drought stress leaves are less or even insufficiently supplied with sulfate. At the same time roots show ample presence of sulfur for the synthesis of organic compounds, either because of re-allocation of sulfur from the leaves, decreased sulfate transport to the shoot or less likely increased sulfate uptake.

**Fig. 6:**
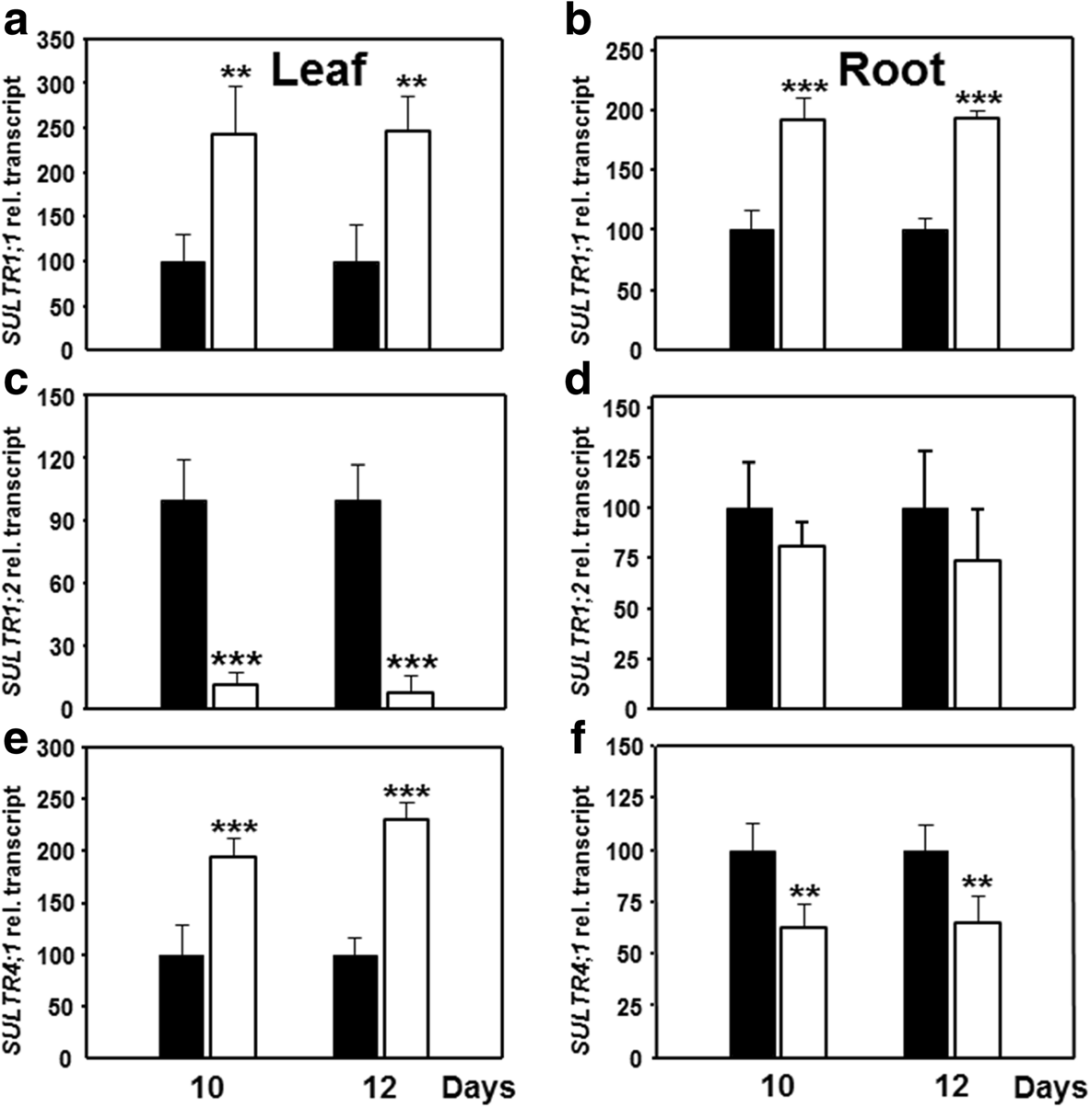
Impact of drought stress on transcription of sulfate transporters leaves and roots of maize. Transcript steady state levels of three genes encoding for maize sulfate transporters *SULTR1;1* (**a**, **b**), *SULTR1,2* (**c**, **d**) and *SULTR4;1* (**e**, **f**) in leaves (**a**, **c**, **e**) and roots (**b**, **d**, **f**) of control (*black*) and drought-stressed plants (*white*). Data are means ± SD of three individual replicates. Asterisks indicates statistical differences as determined by the unpaired *t*-test (*, 0.05 ≥ *p* > 0.01; **, 0.01 ≥ *p* > 0.001; ***, *p* ≤ 0.001)

These findings prompted us to investigate the sulfate uptake mechanisms during drought stress in an organ specific manner. Since direct sulfate uptake experiments are not possible in drought stress roots, the expression of *SULTR* genes was determined instead. The levels of *SULTR1;1* mRNA were 2-2.5-fold higher in leaf and root in response to drought, (Fig. 6a, b). Blast search with the Arabidopsis *SULTR1* sequences resulted in identification of the second member of the *SULTR1* family in maize that is named here *SULTR1;2* (GRMZM2G080178). The expression of the maize *SULTR1;2* gene was strongly reduced in leaves but unchanged in roots during drought stress (Fig. 6c, d).

Expression of the gene encoding *SULTR4;1* (ACG29567) that is responsible for release of sulfate from the vacuole in Arabidopsis [38] showed a reciprocal pattern: it was up-regulated in leaf but was down-regulated in roots, strongly indicating mobilization of stored sulfate in leaves and retention in roots cell vacuoles (Fig. 6e, f).

### Sulfur incorporation of leaves during drought

Lowered metabolite steady-state concentrations but elevated expression of genes of sulfate transport and glutathione metabolism in drought-stressed leaves strongly suggested a program to activate the sulfate reduction pathway towards glutathione synthesis. To gain insight into the in vivo situation of these processes the flux of radiolabeled ^35^S-sulfate via the sulfate reduction pathway into cysteine and glutathione was monitored in leaves. Prior to this analysis we demonstrated that re-hydration of the analyzed leaf discs with respect to RWC was insignificant for the time span of the experiment. In contrast, several attempts to feed drought-stressed roots produced inconsistent results due to the problem of substantial re-hydration during the experiment.

**Fig. 7:**
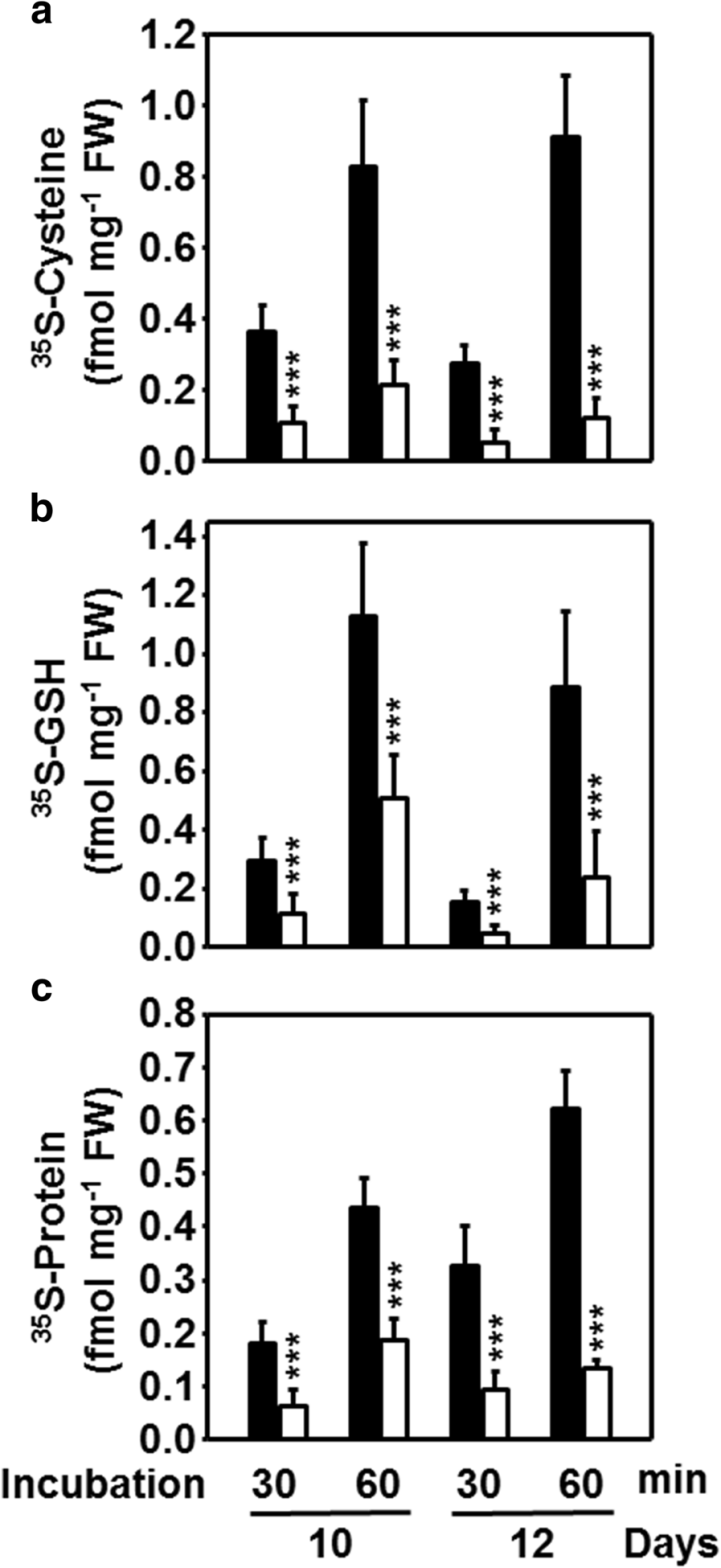
Incorporation of sulfate into cysteine (**a**), glutathione (**b**) and proteins (**c**) in leaves of drought-stressed maize. Leaf pieces of plants with continuous (*black*) or no supply of water (*white*) for 10 and 12 days were first rehydrated in water and subsequently floated for 30 or 60 min on [^35^S]-sulfate containing medium according to [39]. Proteins and metabolites were extracted and [^35^S]-label was quantified in the different fraction by scintillation counting. Data are means ± SD of eight individual replicates. Asterisks indicates statistical differences as determined by the unpaired *t*-test (*, 0.05 ≥ *p* > 0.01; **, 0.01 ≥ *p* > 0.001; ***, *p* ≤ 0.001)

In control leaves the incorporation of ^35^S from ^35^S-sulfate into cysteine approximately doubled from 30 to 60 min, both on day 10 and 12 (Fig. 7a). The vast majority of synthesized cysteine in unstressed leaves from Arabidopsis is channeled in similar amounts into either glutathione or proteins [39]. In maize the incorporation of [^35^S]-label from cysteine into glutathione was increased 3- to 4-fold between 30 and 60 min on day 10 and 12, while the transfer into the protein fraction doubled in controls (Fig. 7b, c). Feeding of leaf discs from drought-stressed maize plants revealed significantly decreased incorporation of ^35^S into cysteine (70–80 %), glutathione (65–70 %) and protein (65–73 %) relative to control at each time point. Despite the enhanced oxidative stress under drought no increased channeling of reduced sulfur into the glutathione pool was observed. The time course patterns at these lowered levels were very much like in the controls, all together indicating that the experimental system worked reliably with control and drought-stressed leaf material. Taken together, the reduced flux through the pathway was consistent with the lowered thiol contents as consequence of specific limitation of sulfate availability and corresponded to the decreased growth of leaves under drought stress.

### Impact of drought on root-to-shoot sulfate transport capacity

**Fig. 8:**
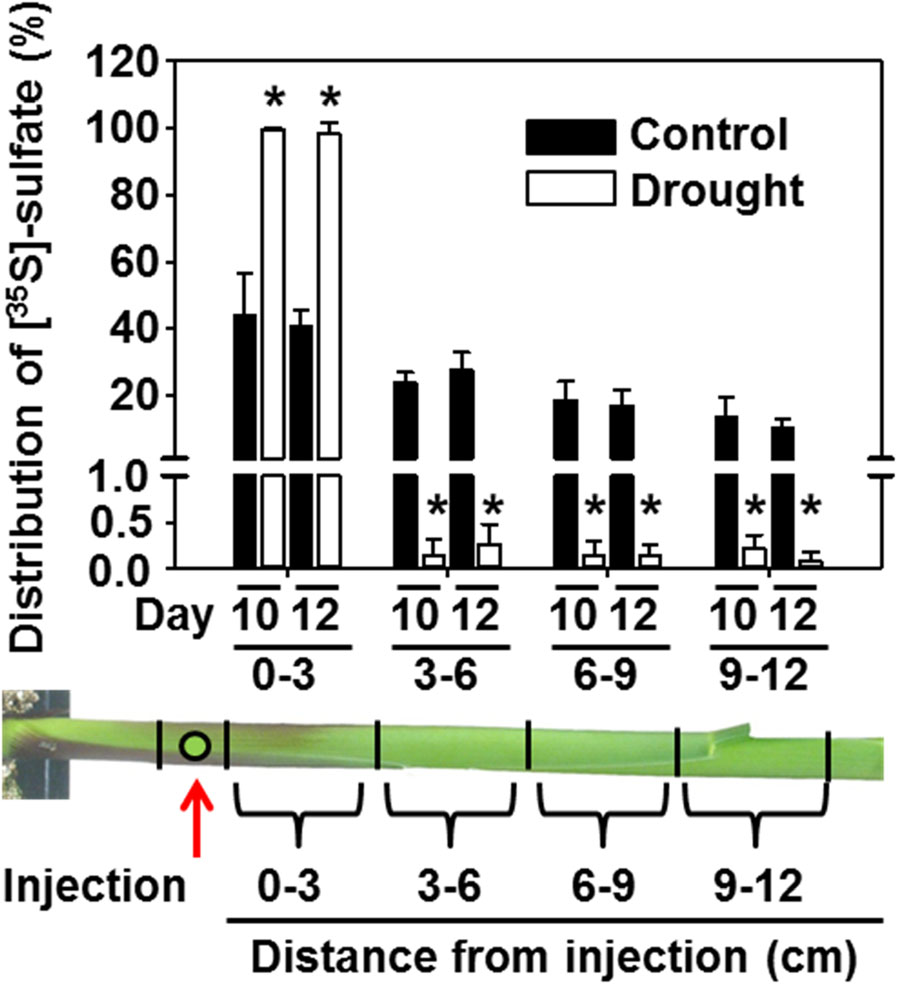
Transport of sulfate within the shoot of drought-stressed maize. Distribution of sulfate within stem of control (*black*) and drought-stressed plants (*white*) 1 min after injection of [^35^S]-sulfate at indicated site. Data are means ± SD of six individual replicates. Asterisks indicates statistical differences as determined by the unpaired *t*-test (*, *p* ≤ 0.001)

The significant accumulation of sulfate in the still well growing roots of drought-stressed plants prompted us to test if decreased root to shoot transport contributed to the specific accumulation of sulfate in this organ upon drought. Transport of vasculature injected 35S-sulfate was significantly decreased in plants subjected to drought for 10 or 12 day when compared to control plants (Fig. 8). This significant decrease in the sulfate transport capacity of drought stressed maize is in full agreement with the observed stomatal closure, since transpiration via the stomata is a known driver of the transport rate of solutes in the xylem.

## Discussion

### Differential regulation of sulfur metabolism in leaves and roots upon drought

Drought has become the most important environmental stress affecting productivity of field crops. Maize is one of the most intensively breed staple crops, but despite these efforts, the sensitivity of high yielding maize varieties to drought stress has been increased in the last few years [40]. The morphological and physiological responses that lead to drought tolerance are based on numerous genetic loci of which only few have been functionally identified [17]. In this context several recent discoveries point to an unexpected, yet important role of sulfur metabolism in the formation of drought stress tolerance (reviewed in [15]).

Despite the compelling evidence of an important role of sulfur-related compounds and processes during drought stress the metabolism of sulfur has not been investigated in this respect. Previous studies on sulfate uptake, reduction and integration into sulfur-containing amino acids and other compounds in Arabidopsis and maize mostly focused on mineral and heavy metal stress (reviewed in [24, 27]). In view of these observations the metabolism of sulfur was investigated in maize plants that were exposed to drought stress until the appearance of several typical traits and markers: shift of the root-to-shoot ratio, elevated H_2_O_2_ levels, enhanced oxidation of glutathione and increased proline concentrations. Care was taken that the stressed plants could fully recover upon addition of water. The major previously unknown findings were the up-regulation of genes and/or enzymes activities related to sulfate uptake and metabolism and the fact that leaves and roots were differently effective in coping with the stress situation. Drought sensing and the appearance of oxidative stress took place in both organs as evidenced by proline formation and glutathione redox state. However, only the roots were found to be able to effectively raise their cysteine and glutathione contents and manage to continue to grow, while leaves had lowered glutathione levels and showed decreased flux from sulfate into cysteine in parallel to growth arrest.

### Specific down-regulation of SERAT activity causes decreased cysteine and glutathione production upon drought in leaves

The mechanistic explanation for the decreased flux of sulfate into cysteine in drought-stressed maize leaves is the low availability of sulfide and the significant down-regulation of the cysteine synthesis-limiting SERAT activity (Fig. 4a, g) [41, 42]. SERAT provides the carbon and nitrogen containing backbone for fixation of reduced sulfur and its activity is highly controlled in plants by formation of the cysteine synthase complex [43, 44]. Interaction of SERAT with OAS-TL within the cysteine synthase complex regulates the cysteine feedback sensitivity of SERAT [43, 44], thus, SERAT and OAS-TL transcription and protein abundance are hardly regulated in response to sulfate deficiency [24]. Information on regulation of SERAT activity in response to other environmental stresses is scarce, in particular in maize, and absent for drought stress. However, short term application of oxidative stress-inducing menadione to the reference plant Arabidopsis changed the flux of carbon within primary metabolism resulting in a switch from anabolic to catabolic metabolism. Surprisingly, this switch did not affect the carbon flux into cysteine [45], due to the strong transcriptional induction of the major SERATs (SERAT1;1, SERAT2;1 and SERAT2;2) by menadione-induced ROS [46]. This specific activation of cysteine biosynthesis can be interpreted as a response of plant cells to cope with high ROS levels, since glutathione synthesis is limited by cysteine provision in leaves [31].

Plants under drought stress tend to enhance the level of ROS [13, 47, 48]. Consequent increases of the ratio of GSSG to GSH and GR gene expression and activity have often been reported (reviewed in [14, 15]). These changes were also observed under the drought stress conditions applied here (Fig. 2). The increase of the GSSG/GSH ratio in both leaves and roots indicate severe oxidative stress. To counteract the production of GSSG the GR transcript level and the enzymatic activity in leaves increased, confirming the important role of GR in ROS detoxification [48, 49]. However, in the analyzed stages of drought stress, flux of sulfur into cysteine was depleted, which concomitantly depleted also GSH levels. As observed here for maize leaves, drought stress resulted in decreased leaf glutathione levels in *Cochlearia atlantica* [50], *Sporobolus stapfianus* [51], wheat [52] and rice seedlings [53, 54], while glutathione accumulated in grasses [55] and sunflower seedling [56]. The specific mechanisms for the alteration of glutathione levels remain unknown in these plants and might be dependent on severity and duration of stress. A known trigger of stress-induced GSH biosynthesis is the stimulation of GSH1 activity by redox-regulation. Stress-induced oxidation of GSH1 will activate the enzyme and allows counteracting the oxidizing milieu by *de novo* synthesis of reduced glutathione [57, 58]. This well-established enzymatic feed-back mechanism for glutathione biosynthesis cannot refill the glutathione pool in drought stressed maize leaves, since GSH1 activity is limited by cysteine supply upon the here applied drought stress condition. This result is consistent with the known rate-limiting function of cysteine in leaves for GSH synthesis [31]. Also the transcriptional induction of the *GSH1* gene was not sufficient to trigger flux of sulfur into glutathione in drought stressed maize leaves (Fig. 3g). The limitation of GSH synthesis by cysteine in a stress situation that causes significant ROS formation (Fig. 2c) is counterintuitive but evident from flux analysis (Fig. 7). The most likely explanation for the surprising down-regulation of cysteine synthesis (Figs. 3e, 4a, g, 7) in leaves is that sulfur accumulated in the root to maintain growth and ROS detoxification upon drought.

### Sulfate accumulates and is actively metabolized in drought stressed roots

Since an additional major difference under drought stress was found to be the high sulfate concentration in roots as compared to leaves (Fig. 5c, d), the availability of sulfate for reduction and synthesis of cysteine appears to contribute to the better performance of roots under drought stress (Fig. 1e, f). Enhanced sulfate reduction in roots is strongly indicated by higher sulfide steady state levels. The enhanced sulfide levels will activate endogenous in vivo SERAT activity without affecting extractable SERAT amount due to formation of the cysteine synthase complex [44, 59]. Besides the beneficial impact of enhanced cysteine concentration in drought-stressed roots for ABA production [4], methionine synthesis, translation and consequently growth (reviewed in [15, 60]), roots were able to ameliorate their glutathione redox state by increasing total glutathione [61]. Significant transcriptional induction of the *GSH1* gene in roots likely contributed to the increase of glutathione in roots. According to the Nernst-equation this specific increase of GSH will contribute to a more reduced cellular environment of the root [61]. This is particularly important since the GSSG/GSH ratio also increased in roots (Fig. 2e). GSSG might increase in roots due to export into the vacuole [62], unchanged GR activity (Fig. 2i) or limited supply of the electron donor NADPH for activities of GR and thioredoxins that constitute a functional backup for GR [16].

The origin of the specific sulfate accumulation in roots during water withdrawal could be due to (1) re-translocation of sulfur from the shoot to the root, (2) lowered transport from the root to the shoot or (3) enhanced sulfate uptake: (1) Recycling of nutrients during stress is mediated in eukaryotes by autophagy. Indeed, autophagy modulates the tolerance towards drought and salt stress and is induced upon osmotic stress by NADPH oxidase-generated ROS [63]. However, induction of autophagy during drought does not imply transport of nutrients from the shoots to the roots, since it is also important for intracellular mobilization of nutrients and clearance of damaged intracellular structures [64]. Considerable recycling of sulfate from the shoot to the root might be also facilitated by the specific up-regulation of the *SULTR4;1* in drought-stressed maize leaves. In Arabidopsis SULTR4;1 remobilizes long-term stored sulfate from the vacuole of leaf cells [38]. (2) In line with the determined lowered sulfate transport capacity from the root to the shoot during drought is the drought-induced closure of stomata. Stomatal closure is supposed to affect root-to-shoot sulfate transport by decreasing the transpiration stream in the xylem, the main road for sulfate transport to the shoot [24, 38]. Furthermore, the specific transcriptional down-regulation of the vacuolar sulfate efflux transporter *SULTR4;1* in roots might add to observed accumulation of sulfate in drought-stressed maize roots, since *SULTR4;1* down-regulation will decrease sulfate efflux from the vacuole. (3) In general, water stress conditions lower diffusion rates of minerals at low soil water status. The transcriptional induction of *SULTR1;1* in drought-stressed maize roots must therefore not necessarily result in higher sulfate uptake rates. Uptake of total nitrogen and potassium was not increased in roots of maize, rice and soybean upon drought stress [65].

## Conclusions

Leaves and roots showed significant transcriptional up-regulation of glutathione synthesis (GSH1) and reduction (GR) in order to counteract the drought stress-induced reactive oxygen species formation. However, we demonstrated that the flux of sulfur from sulfate into cysteine and glutathione is low in leaves of drought stressed plants, ultimately resulting in enhanced oxidative stress, which together contribute to growth arrest of leaves. The low flux of sulfur into glutathione is a result of decreased SERAT activity and low sulfate availability. In contrast, roots accumulate sulfate to support sulfide, cysteine and glutathione formation, and maintain growth. The results evidence a significant and organ-specific impact of drought upon sulfate assimilation in the staple crop maize. We conclude that the antagonistic regulation of sulfur metabolism in leaves and roots enables a successful drought stress response at the whole plant level. These findings add sulfur metabolism as a new player in the drought stress response of maize and uncover a new target to improve drought stress resistance. The results set the stage to study the role of sulfur-metabolism related processes and signals as drivers for drought-induced developmental plasticity.

## Methods

### Plant growth and drought stress

Maize (*Zea mays* L) hybrid Severo seeds were obtained from KWS Germany for drought stress experiments. Seeds were sown individually in each pot containing 100 % vermiculite media and grown in long day conditions with 16 h/8 h day/night cycle at a light intensity of 300 μmol m^-2^ s^-1^, 22 °C/20 °C and 50 % humidity.

One week after sowing, plants were watered three times per week with ½ Hoagland solution (2.5 mM Ca(NO_3_)_2_, 2.5 mM KNO_3_, 0.5 mM MgSO_4_, 0.5 mM KH_2_PO_4_, 40 mM Fe-EDTA, 25 mM H_3_BO_3_, 2.25 mM MnCl_2_, 1.9 mM ZnSO_4_, 0.15 mM CuSO_4_, and 0.05 mM (NH_4_)_6_MO_7_O_24_, pH 5.8). Two weeks after sowing in half of the plants irrigation was withheld for 7, 10 and 12 days (drought stress) while the remaining plants were supplied three times per week (control treatment).

### Measurement of the relative water content (RWC)

Measurement of the relative water content (RWC) was performed according to [66]. Briefly, individual leaves were removed from the stem using scissors and fresh weight (FW) was recorded immediately. The leaves were then incubated in distilled water for at least 4 h at 4 °C in the dark, blotted dried and then turgid weight (TW) was measured. Finally, dry weight (DW) was determined after incubation at 80 °C for 48 h in the oven. The relative water content (RWC) was calculated with the following formula as described by Jones (2007): RWC (%) = [(FW - DW)/ (TW - DW)] * 100.

### Measurement of stomatal aperture

Quantification of stomatal aperture was performed by doing a leaf imprint on a droplet of superglue on microscope slide. Truncated leaf discs from control and drought-stressed plants were placed immediately on the slide with cuticle side up and the lower epidermis down on the glue droplet. The leaf discs were then gently pressed so that the lower part of the leaf stuck to the slide and afterwards with the help of forceps, leaf disc was removed forming an image on the slide. Stomatal aperture was analyzed with microscope and Image J (https:\\imagej.nih.gov). The stomatal aperture refers to the distance between the outer borders of stoma cells.

### Determination of metabolites and in situ staining of H_2_O_2_

Thiols, amino acids, OAS, anions were determined from leaves and roots of control and drought-stressed plants according to [67]. Total sulfur contents were quantified as described by [37]. Sulfide contents were determined using the procedure according to [68]. For calculation of GSH/GSSG ratios the extraction of GSH was performed as explained in [69].

In situ staining of H_2_O_2_ in leaves of control and drought-stressed plants was performed according to [70] by vacuum infiltration of a freshly prepared 3,3′-diaminobenzidine (DAB) solution (1.68 mg/ml in dH_2_O; pH 3.8) followed by incubation for 24 h at room temperature. After discoloration of chlorophyll with pure ethanol, images were recorded with the color LCD 320 FX camera (Leica) with 2.5x magnification.

### Determination of enzymatic activities

The extraction and quantification of soluble proteins from the leaf and root of control and drought-stressed maize plant was performed as described by [39]. Glutathione reductase activity was determined according to [16] using 20 μg of soluble proteins in a total volume of 250 μl reaction mixture containing 100 mM K_2_HPO_4_/KH_2_PO_4_ pH 7.4, 1 mM ethylene diamine tetracetate EDTA together with 750 μM dithio-nitrobenzoic acid, 200 μM NADPH and 400 μM GSSG.

Enzymatic activities of SERAT and OAS-TL were determined by quantification of the reaction product cysteine according to [67] and [71], respectively. The reactions were performed in an assay volume of 0.1 ml containing 1 μg of soluble leaf-proteins for OAS-TL and 50 μg of soluble leaf-proteins for SERAT activity. The reaction was started by the addition of master mix to the crude extract and allowed to proceed for up to at 25 min at 25 °C.

### RNA isolation and qRT-PCR analysis

Approximately 100 mg of leaf and root tissue was used for total RNA extraction using RNeasy Plant Mini Kit and RNase free DNAse Kit and PeqGOLD total RNA kit (Qiagen, and Peqlab, Germany). Synthesis of cDNA from total RNA extract was performed with RevertAid™ H Minus First Strand cDNA Synthesis Kit (Thermo Scientific, Germany). The qRT-PCR reaction was performed with 1 μg cDNA and 2.5 pmol of each specific primer and was mixed with 6.25 μl SYBR solution from Rotor Gene Sybr Green PCR Kit (Qiagen). The reaction took place in the Rotor-Gene Q cycler (Qiagen, Germany) according to the manufacturer’s protocol. Actin or γ-tubulin served as reference genes for normalization of qRT-PCR data in leaves or roots, respectively. Primers for qRT-PCR from maize are listed in Additional file 4: Table S1.

### Determination of incorporation of ^35^S into thiols and protein of maize leaves

Approximately 30 mg of leaf discs of comparable sizes were cut from the control and drought-stressed plants and rehydrated in dH_2_O for 10 min, This was followed by incubation in the ^35^S-sulfate labeling solution (in ½ Hoagland medium) for 30 and 60 min with a total of 0.502 mM sulfate containing 125 nM ^35^SO_4_^2-^ on a horizontal shaker at 60 rpm in the light (17 μE). After incubation on ^35^SO_4_^2-^ labeling solution, the leaf pieces were washed twice with nonradioactive ½ Hoagland medium, dried on paper towel and immediately frozen in liquid nitrogen. Homogenization of the radiolabeled leaf samples was performed using the Bio101 ThermoSavant Fast Prep system (Qbiogene) according to the manufacturer’s instructions. The extraction, derivatization and detection of metabolites were performed as described by [39].

### Quantification of ^35^S-sulfate transport within the stem of drought stressed maize

[35S]-sulfate (0.75 fmol) was injected into stems of control and drought stressed plants approximately 3.5 cm above the soil level of stems. Plants were illuminated with constant light for 1 min and harvested by simultaneous cutting of four stem segments (each 3 cm) 0.5 cm above the injection site. The resulting segments of the stem were separately grounded in liquid nitrogen and the radioactivity was quantified by scintillation counting as described in [39].

### Statistical analyses

Means of different data sets were analyzed for statistical significance using unpaired *t*-test or ANOVA test. Constant variance and normal distribution of data were checked with SigmaStat 12.0 prior to statistical analysis. The Mann-Whitney rank sum test was used to analyze samples that did not follow normal Gaussian distribution. Asterisks in all figures indicate the significance: *, 0.05 ≥ *p* > 0.01; **, 0.01 ≥ *p* > 0.001; ***, *p* ≤ 0.001.

## Additional files

Additional file 1: Figure S1. Stomatal aperture of drought stressed maize plants. Quantification of stomatal aperture of control (black) and water-restricted (white) maize leaves at indicated time points. Data are means ± SD of 35 individual replicates. Asterisks indicates statistical differences as determined by the unpaired *t*-test (*, 0.05 ≥ *p* > 0.01; **, 0.01 ≥ *p* > 0.001; ***, *p* ≤ 0.001). (PDF 223 kb)
Additional file 2: Figure S2. Alignment of cytosolic glutathione reductase 1 (GR1; At3g24710) and the plastid and mitochondria localized GR2 (At2g54660) from *Arabidopsis thaliana* with the single identified homologous GR protein sequence from maize (GenBank accession no. AJ006055) with the CLUSTALW software. The alignment of these sequences showed sequence identity of maize GR of approximately 52 % with GR1 and 78 % with GR2 from Arabidopsis. (PDF 198 kb)
Additional file 3: Figure S3. Steady state levels of glutamate and glycine in roots and shoots of drought stressed maize plants. A-B) Steady state levels of glutamate (A) and glycine (B) in leaves and roots of maize plants with sufficient (black) and restricted (white) water supply. Data are means ± SD of five individual replicates. Asterisks indicates statistical differences as determined by the unpaired *t*-test (*,*p* ≤ 0.05). (PDF 155 kb)
Additional file 4: Table S1. Accession number, genome annotations (http://www.maizegdb.org/) and primers used for quantification of transcript steady levels by qRT-PCR of maize genes addressed in this study. (PDF 202 kb)

## Abbreviations

ABA: Abscisic acid
APR: Adenosine-5-phosphosulfate reductase
ATPS: ATP-sulfurylase
DAB: 3,3′-Diaminobenzidine
GR: Glutathione reductase
GSH: Reduced glutathione
GSSG: Oxidized glutathione
H_2_O_2_: Hydrogen peroxide
OAS: *O*-acetylserine
OASTL: *O*-acetylserine(thiol)lyase redox
ROS: Reactive oxygen species
RWC: Relative water content
SERAT: Serine acetyltransferase
SiR: Sulfite reductase
SULTR: Sulfate transporters
γ-EC: γ-glutamylcysteine
