# Supplementary material for: Drought stress in maize causes differential acclimation responses of glutathione and sulfur metabolism in leaves and roots
Source: BMC Plant Biol. 2016 Nov 9;16:247. doi: 10.1186/s12870-016-0940-z (PMC5103438; doi:10.1186/s12870-016-0940-z)
Supplement: Additional file 1: Figure S1. — Stomatal aperture of drought stressed maize plants. Quantification of stomatal aperture of control (black) and water-restricted (white) maize leaves at indicated time points. Data are means ± SD of 35 individual replicates. Asterisks indicates statistical differences as determined by the unpaired t-test (*, 0.05 ≥ p > 0.01; **, 0.01 ≥ p > 0.001; ***, p ≤ 0.001). (PDF 223 kb) [file 12870_2016_940_MOESM1_ESM.pdf]

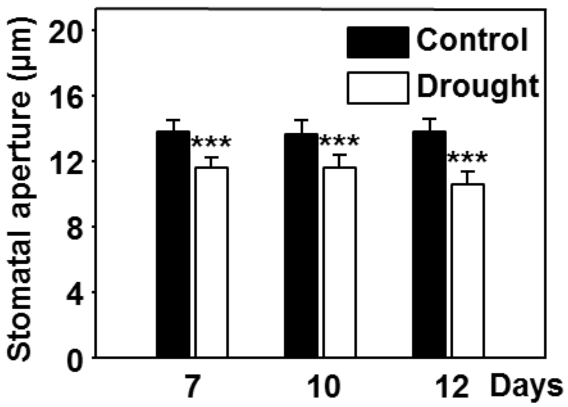

Supplementary Figure 1. Stomatal aperture of drought stressed maize plants.

Quantification of stomatal aperture of control (black) and water-restricted (white) maize leaves at indicated time points. Data are means ± SD of 35 individual replicates. Asterisks indicates statistical differences as determined by the unpaired t-test (*, $0.05 \geq p > 0.01$; **, $0.01 \geq p > 0.001$; ***, $p \leq 0.001$).
