# Supplementary material for: Drought stress in maize causes differential acclimation responses of glutathione and sulfur metabolism in leaves and roots
Source: BMC Plant Biol. 2016 Nov 9;16:247. doi: 10.1186/s12870-016-0940-z (PMC5103438; doi:10.1186/s12870-016-0940-z)
Supplement: Additional file 2: Figure S2. — Alignment of cytosolic glutathione reductase 1 (GR1; At3g24710) and the plastid and mitochondria localized GR2 (At2g54660) from Arabidopsis thaliana with the single identified homologous GR protein sequence from maize (GenBank accession no. AJ006055) with the CLUSTALW software. The alignment of these sequences showed sequence identity of maize GR of approximately 52 % with GR1 and 78 % with GR2 from Arabidopsis. (PDF 198 kb) [file 12870_2016_940_MOESM2_ESM.pdf]

```
MaizeGR             MAAHATLPFSCSS---TLQTLTRTL--SSRGAHQLRGGFLRLP----SLAAL-PRLAHPC 50
ArabidopsisGR2      MASTPKLTSTISSSSPSLQFLCKKLPIAIHLPSSSSSSFLSLPKTLTSLYSLRPRIALLS 60
ArabidopsisGR1      MARKMLVDGEIDK----------------------------------------------- 13
                    **    :    ..

MaizeGR             RRHVSAS-----AAAAPNGASVEGEYDYDLFTIGAGSGGVRASRFASALYGSRVAICEMP 105
ArabidopsisGR2      NHRYYHSRRFSVCASTDNGAESDRHYDFDLFTIGAGSGGVRASRFATS-FGASAAVCELP 119
ArabidopsisGR1      ------------VAADEANAT---HYDFDLFVIGAGSGGVRAARFSAN-HGAKVGICELP 57
                                 *:   .*    .**:***.**********:**::  .*: ..:**:*

MaizeGR             FATIASDELGGLGGTCVLRGCVPKKLLVYASKYSHEFEESRGFGWTYETDPKHDWSTLIA 165
ArabidopsisGR2      FSTISSDTAGGVGGTCVLRGCVPKKLLVYASKYSHEFEDSHGFGWKYETEPSHDWTTLIA 179
ArabidopsisGR1      FHPISSEEIGGVGGTCVIRGCVPKKILVYGATYGGELEDAKNYGWEINEKVDFTWKKLLQ 117
                    * .*:*:  **:*****:*******:***.:.*. *:*:::.:**  : . .. *..*:

MaizeGR             NKNTELQRLVGIYRNILNNAGVTLIEGRGKIVDPHTVSV---NG--KLYTAKHILVSVGG 220
ArabidopsisGR2      NKNAELQRLTGIYKNILSKANVKLIEGRGKVIDPHTVDV---DG--KIYTTRNILIAVGG 234
ArabidopsisGR1      KKTDEILRLNNIYKRLLANAAVKLYEGEGRVVGPNEVEVRQIDGTKISYTAKHILIATGS 177
                    :*. *: ** .**:.:* :* *.* **.*:::.*: *.*   :*    **:::**::.*.

MaizeGR             RPSMPDIPGIEHVIDSDAALDLPSKPEKIAIVGGGYIALEFAGIFNGLKSEVHVFIRQKK 280
ArabidopsisGR2      RPFIPDIPGKEFAIDSDAALDLPSKPKKIAIVGGGYIALEFAGIFNGLNCEVHVFIRQKK 294
ArabidopsisGR1      RAQKPNIPGHELAITSDEALSLEEFPKRAIVLGGGYIAVEFASIWRGMGATVDLFFRKEL 237
                    *.  *:*** * .* ** **.* . *::  ::******:***.*:.*: . *.:*:*::

MaizeGR             VLRGFDEEVRDFVAEQMSLRGITFHTEQSPQAITKSNDGLLSLKTNKENFGGFSHVMFAT 340
ArabidopsisGR2      VLRGFDEDVRDFVGEQMSLRGIEFHTEESPEAIIKAGDGSFSLKTSKGTVEGFSHVMFAT 354
ArabidopsisGR1      PLRGFDDEMRALVARNLEGRGVNLHPQTSLTQLTKTDQG-IKVISSHGEEFVADVVLFAT 296
                     *****:::* :*..::. **: :*.: *   : *:.:* :.: :.:      . *:***

MaizeGR             GRRPNSKNLGLEAVGVEMDKNGAIVVDEYSRTSVDSIWAVGDVTNRVNLTPVALMEGGAF 400
ArabidopsisGR2      GRKPNTKNLGLENVGVKMAKNGAIEVDEYSQTSVPSIWAVGDVTDRINLTPVALMEGGAL 414
ArabidopsisGR1      GRSPNTKRLNLEAVGVELDQAGAVKVDEYSRTNIPSIWAVGDATNRINLTPVALMEATCF 356
                    ** **:*.*.** ***:: : **: *****:*.: *******.*:*:*********. .:

MaizeGR             AKTVFGNEPTKPDYRAIPSAVFSQPPIGQVGLTEEQAIEEY-GDVDVFVANFRPLKATLS 459
ArabidopsisGR2      AKTLFQNEPTKPDYRAVPCAVFSQPPIGTVGLTEEQAIEQY-GDVDVYTSNFRPLKATLS 473
ArabidopsisGR1      ANTAFGGKPTKAEYSNVACAVFCIPPLAVVGLSEEEAVEQATGDILVFTSGFNPMKNTIS 416
                    *:* * .:***.:*  :..***. **:. ***:**:*:*:  **: *:.:.*.*:* *:*

MaizeGR             GLPDRVLMKILVCATSNKVVGVHMCGDDAPEIIQGIAIAVKAGLTKQDFDATIGIHPTSA 519
ArabidopsisGR2      GLPDRVFMKLIVCANTNKVLGVHMCGEDSPEIIQGFGVAVKAGLTKADFDATVGVHPTAA 533
ArabidopsisGR1      GRQEKTLMKLIVDEKSDKVIGASMCGPDAAEIMQGIAIALKCGATKAQFDSTVGIHPSSA 476
                    *  ::.:**::*  .::**:*. *** *:.**:**:.:*:*.* ** :**:*:*:**::*

MaizeGR             EEFVTMRSPTRKIRKSSTDQVESKDEVVSKQ- 550
ArabidopsisGR2      EEFVTMRAPTRKFRKDSSEGKASPEAKTAAGV 565
ArabidopsisGR1      EEFVTMRSVTRRIAHKPKPKTNL--------- 499
                    *******: **:: :...
```

Supplementary Figure 2. Alignment of cytosolic glutathione reductase 1 (GR1; At3g24710) and the plastid and mitochondria localized GR2 (At2g54660) from *Arabidopsis thaliana* with the single identified homologous GR protein sequence from maize (GenBank accession no. AJ006055) with the CLUSTALW software. The alignment of these sequences showed sequence identity of maize GR of approximately 52% with GR1 and 78% with GR2 from Arabidopsis.
