# Supplementary material for: Drought stress in maize causes differential acclimation responses of glutathione and sulfur metabolism in leaves and roots
Source: BMC Plant Biol. 2016 Nov 9;16:247. doi: 10.1186/s12870-016-0940-z (PMC5103438; doi:10.1186/s12870-016-0940-z)
Supplement: Additional file 3: Figure S3. — Steady state levels of glutamate and glycine in roots and shoots of drought stressed maize plants. A-B) Steady state levels of glutamate (A) and glycine (B) in leaves and roots of maize plants with sufficient (black) and restricted (white) water supply. Data are means ± SD of five individual replicates. Asterisks indicates statistical differences as determined by the unpaired t-test (*,p ≤ 0.05). (PDF 155 kb) [file 12870_2016_940_MOESM3_ESM.pdf]

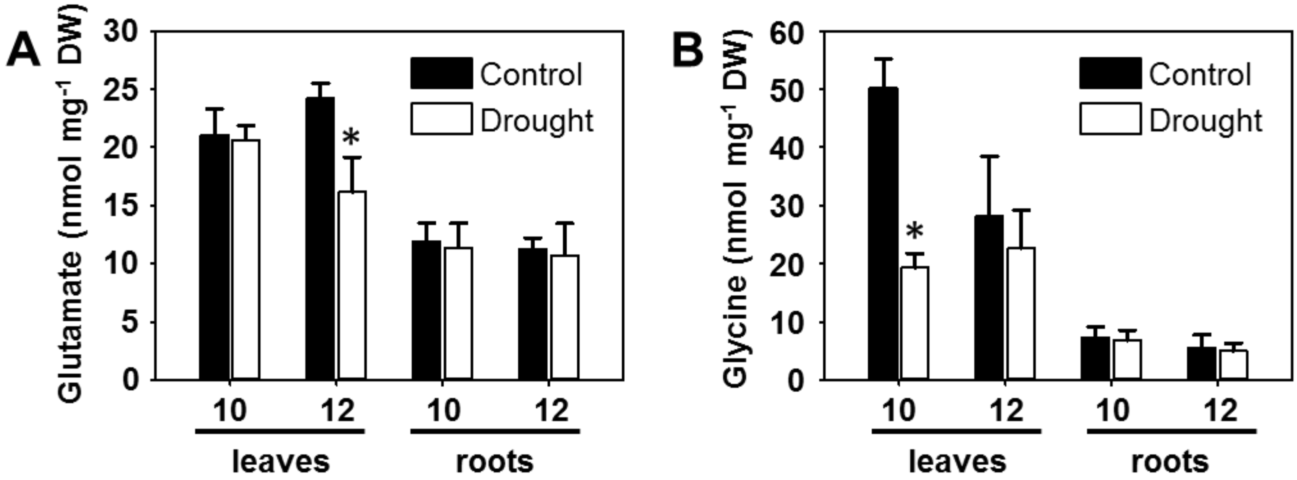

Supplementary Figure 3: Steady state levels of glutamate and glycine in roots and shoots of drought stressed maize plants

A-B) Steady state levels of glutamate (A) and glycine (B) in leaves and roots of maize plants with sufficient (black) and restricted (white) water supply. Data are means ± SD of five individual replicates. Asterisks indicates statistical differences as determined by the unpaired t-test (*,p $\leq$ 0.05)
