# Supplementary material for: Drought stress in maize causes differential acclimation responses of glutathione and sulfur metabolism in leaves and roots
Source: BMC Plant Biol. 2016 Nov 9;16:247. doi: 10.1186/s12870-016-0940-z (PMC5103438; doi:10.1186/s12870-016-0940-z)
Supplement: Additional file 4: Table S1. — Accession number, genome annotations (http://www.maizegdb.org/) and primers used for quantification of transcript steady levels by qRT-PCR of maize genes addressed in this study. (PDF 202 kb) [file 12870_2016_940_MOESM4_ESM.pdf]

Supplementary table 1

Accession number, genome annotations (http://www.maizegdb.org/) and primers used for quantification of transcript steady levels by qRT-PCR of maize genes addressed in this study.

Gene name	Accession number	Maize genome annotation	Name of primer	Sequence
Actin	J01238	GRMZM2G126010	Actin_for<br>Actin_rev	CTCAACCCCAAGGCCAACAGAGAG<br>GGCTCACACCATCACCTGAATCCA
APS reductase	AJ295032	-	APR_for<br>APR_rev	GTCCAGGTTGATCCTTCCTT<br>GTCAATGTTGCCCTTGTGGA
ATP sulfurylase 1	-	GRMZM2G051270	ATPS1_for<br>ATPS1_rev	CAGATGATGTGCCTCTTAGTTGGAG<br>GCATTGGAGAGGGAAAGATCGCAAC
ATP sulfurylase 2	-	GRMZM2G158147	ATPS2_for<br>ATPS2_rev	TGATACAGTAGCAAAGGAGATGGCC<br>GAAACCGTCTGGAGGATTCTCTCC
ATP sulfurylase 3	-	GRMZM2G149952	ATPS3_for<br>ATPS3_rev	CCTGTTCTTCTGCTCCATCCACTG<br>CAGTTGATTCTGGGTTGAGGACACC
Glutathione reductase	AJ006055	GRMZM2G172322	GR_for<br>GR_rev	CATTTGGGCTGTTGGAGATG<br>TGACGTAGCGCACACAAGAA
γ-EC synthetase	AJ302783	GRMZM2G020096	GSH1_for<br>GSH1-rev	AGCTTAAGGAGCCATATCTGGACAG<br>GCATAGTCCACATATTGCTCAAACCC
SULTR1;1	AF355602	GRMZM2G159632	SULTR1;1_for<br>SULTR1;1_rev	TCCTGGCATTCCTTCTGGTT<br>GAGGGCTGCAAATGTTCTTC
SULTR1;2	EU974789	GRMZM2G080178	SULTR1;2_for<br>SULTR1;2_rev	GGCGTGGTTTTCAAGTCTGT<br>ATCTTCTTCGTCCGTCAACC
SULTR4;1	ACG29567	GRMZM2G068212	SULTR4;1_for<br>SULTR4;1_rev	CTACATCAAAGACAGGTTGCGTGAG<br>GTA ACAGGGGACATCTCGAGGATCAC
β-tubulin	L10634	GRMZM2G043822	Tub_for<br>Tub_rev	GATTTGCTCCACTGACCTCGCGG<br>CGGAACATAGCAGATGCCGTGAG
